# A focused 35-minute whole body MRI screening protocol for patients with von Hippel-Lindau disease

**DOI:** 10.1186/s13053-019-0121-9

**Published:** 2019-07-29

**Authors:** Anne-Marie Vanbinst, Carola Brussaard, Evelynn Vergauwen, Vera Van Velthoven, Robert Kuijpers, Olaf Michel, Ina Foulon, Anna C. Jansen, Bieke Lefevere, Susanne Bohler, Kathelijn Keymolen, Johan de Mey, Dirk Michielsen, Corina E. Andreescu, Sven Gläsker

**Affiliations:** 1 Department of Radiology, Universitair Ziekenhuis Brussel, Brussels, Belgium; 2 Department of Neurosurgery, Universitair Ziekenhuis Brussel, Laarbeeklaan 101, 1090 Brussels, Belgium; 3 Department of Ophthalmology, Universitair Ziekenhuis Brussel, Brussels, Belgium; 4 Department of Otorhinolaryngology, Universitair Ziekenhuis Brussel, Brussels, Belgium; 5 Department of Pediatrics, Universitair Ziekenhuis Brussel, Brussels, Belgium; 6 Department of Psychology, Universitair Ziekenhuis Brussel, Brussels, Belgium; 7 Department of Genetics, Universitair Ziekenhuis Brussel, Brussels, Belgium; 8 Department of Urology, Universitair Ziekenhuis Brussel, Brussels, Belgium; 9 Department of Endocrinology, Universitair Ziekenhuis Brussel, Brussels, Belgium

**Keywords:** von Hippel-Lindau disease, Gadolinium, Hereditary cancer, Preventive medicine, MRI screening, Whole body MRI

## Abstract

**Background:**

Von Hippel-Lindau (VHL) disease is an autosomal dominantly inherited tumor syndrome. Affected patients develop central nervous system hemangioblastomas and abdominal tumors, among other lesions. Patients undergo an annual clinical screening program including separate magnetic resonance imaging (MRI) of the brain, whole spine and abdomen. Consequently, patients are repeatedly subjected to time-consuming and expensive MRI scans, performed with cumulative Gadolinium injections. We report our experience with a 35-min whole body MRI screening protocol, specifically designed for detection of VHL-associated lesions.

**Methods:**

We designed an MRI protocol dedicated to the typical characteristics of VHL-associated lesions in different imaging sequences, within the time frame of 35 min. Blank imaging of the abdomen is carried out first, followed by abdominal sequences with Gadolinium contrast. Next, the full spine is examined, followed by imaging of the brain. A single dose of contrast used for abdominal imaging is sufficient for further highlighting of spine- and brain lesions, thus limiting the Gadolinium dosage. We used 1.5 Tesla equipment, dealing with fewer artifacts compared to a 3 Tesla system for spine- and abdominal imaging, while preserving acceptable quality for central nervous system images. In addition, imaging on a 1.5 Tesla scanner is slightly faster.

**Results:**

From January 2016 to November 2018, we performed 38 whole body screening MRIs in 18 VHL patients; looking for the most common types of VHL lesions in the abdomen, spine, and brain, both for new lesions and follow-up. The one-step approach MRI examinations lead to 6 surgical interventions for clinically significant or symptomatic hemangioblastomas in the brain and spine. One renal cell carcinoma was treated with radiofrequency ablation. In comparison with previous conventional MRI scans of the same patients, all lesions were visible with the focused protocol.

**Conclusions:**

Annual screening in VHL disease can be done in a rapid, safe and sensitive way by using a dedicated whole body MRI protocol; saving MRI examination time and limiting Gadolinium dose.

## Background

Patients with hereditary tumor syndromes, such as VHL disease, need to undergo regular MRI screening according to international guidelines [1]. The goal of screening is to enable preventive treatment by early detection of new lesions, together with proper follow-up of previously diagnosed tumors. VHL disease is an autosomal dominant genetic disorder caused by germline mutations in the VHL tumor suppressor gene. Affected patients develop central nervous system (CNS) hemangioblastomas (HB), endolymphatic sac and duct tumors (ELST), renal cysts and clear cell renal cell carcinomas (RCC), pheochromocytomas, pancreatic cysts and cystadenomas, and pancreatic neuroendocrine tumors (NET), among other rare tumors. Male patients frequently develop epididymal cystadenomas [2, 3].

Predicting radiological growth velocity of VHL tumors is often a challenging aspect in therapeutic decision making. This is especially true for HB, mainly found in the retina, cerebellum, and spinal cord; and well known for their capricious growth pattern [4–6]. Therefore, timed radiographic evaluation of CNS HB is indispensable. Although ophthalmologic fundoscopy and fluorescein angiography are current standard for detection of retinal HB, they can also be seen on brain MRI if larger than 2 mm in diameter [3, 7]. Nevertheless, retinal HB are not the primary target in a radiologic screening setting.

ELST are uncommon benign tumors of the inner ear, located in the posterolateral temporal bone. These lesions may cause hearing loss progressing to deafness. ELST are found in approximately 3.6–15% of VHL patients, sometimes bilaterally. When detected and treated early, pre-operative hearing level can sometimes be preserved, avoiding deafness [2, 3, 8].

Renal and pancreatic cysts or cystadenomas are benign and usually asymptomatic. However, patients with VHL show an increased risk of developing malignancies; such as RCC, pancreatic NET, and adrenal or extra-adrenal pheochromocytomas [2, 3]. Detection and strict follow-up of RCC are paramount for timed removal, as lesions over 4 cm harbor an increased risk for distant metastasis [9].

According to international recommendations, follow-up imaging in VHL disease is recommended annually or biennially [1]. Most centers perform separate MRIs for each body region (abdomen, brain, spine) subjecting patients to multiple expensive examinations. Furthermore, CNS accumulation of Gadolinium (Gd) based contrast agents has recently drawn scientific and public attention, occurring in almost all patients after more than 5 MRIs [10, 11]. We have previously reported that Gd accumulations can be observed in virtually all patients included in screening program for VHL disease [12].

Our project aims to develop and evaluate a whole body MRI screening protocol focused on the characteristic features of VHL lesions, reducing the number of MRIs and contrast administrations.

## Methods

### Patient selection

Our center serves as a tertiary reference center for VHL patients from Belgium and other, mostly European countries. All included patients underwent the whole body MRI screening protocol. We included patients in our analysis, with (1) previous conventional MRI in other centers, (2) previous whole body MRI in our center (2), or (3) follow-up whole body MRI in our center. Primary outcomes were to describe the protocol and to prove non-inferiority of our whole body MRI protocol to previous conventional MRIs in other institutions. Our local ethics committee approved the study (ref. no. 2017/071).

### MRI screening protocol

**Fig. 1:**
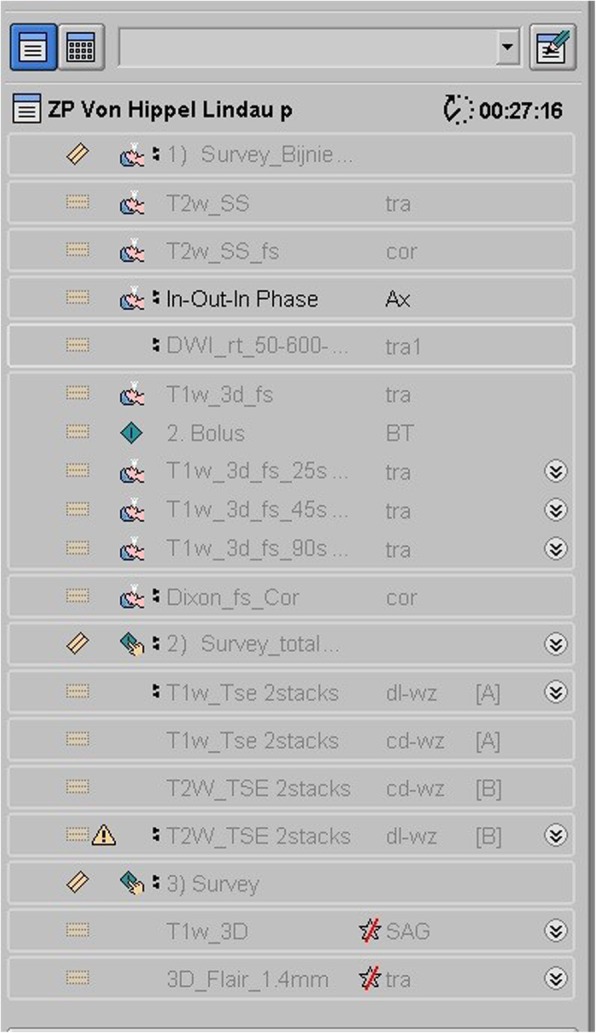
MRI protocol. Screenshot of the examination card we use for our screening protocol, from the MR examination console. Notice the examination time of 27 min and 16 s mentioned on top of the list (time calculated without reconstruction time needed in between sequences)

The imaging protocol was composed using routine sequences. It was explicitly designed to depict VHL-associated lesions by their typical characteristics on specific sequences, and to remove those sequences which are less contributory for detection of VHL lesions (Fig. 1) [13, 14]. Necessary blank imaging of the abdomen is carried out first, followed by the abdominal sequences with Gd contrast. Second, the full spine is examined. Third, the brain is scanned, using 3D-T1 and 3D-FLAIR (fluid attenuated inversion recovery) sequences, which can also be used for neuronavigation in case of later surgery.

The contrast used for abdominal imaging was sufficient to subsequently highlight spine- and brain lesions, thus limiting Gd dosage. Gadoterate meglumine, a macrocyclic ionic contrast agent (Dotarem®, 0.2 ml/kg body weight, with a maximum of 15 ml), was used in all cases.

Aiming for a combined examination of abdomen, brain and spine; all having their own requirements, we preferred to perform the MRIs on a 1.5 T machine (Philips Achieva dStream, Eindhoven, The Netherlands). Especially spine and abdominal imaging are realized faster and with fewer artifacts on a 1.5 T machine compared to a 3 T scanner. Spine images are even of slightly better quality on a 1.5 T machine.

The first stage of the screening protocol (Fig. 1) is dedicated to abdominal imaging.

**Fig. 2:**
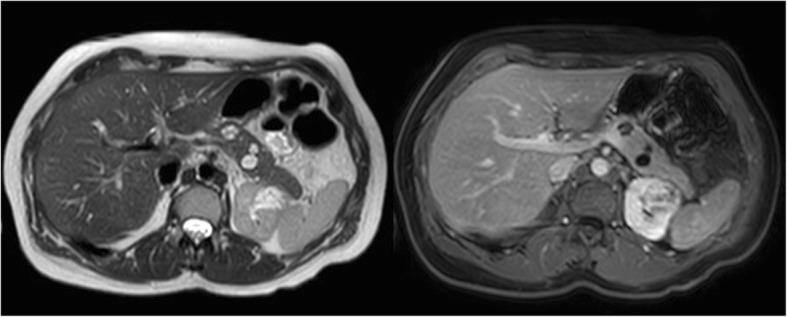
Abdominal stage. Transversal T2 image (left side) and T1 image with fat suppression after intravenous Gd administration in the portal-venous phase. The pancreas contains two small cystadenomas (11 and 13 mm) and a small, simple cyst (4 mm). The upper pole of the left kidney is distorted due to an irregularly enhancing mass (renal cell carcinoma)

T2 transversal images from the dome of the diaphragm to the inferior part of the kidneys, followed by T2 coronal images with fat suppression, provide an overview of the organs; detecting cysts, serous cystadenomas, pancreatic NET, and RCC (Fig. 2). The in-and-out of phase images characterize adrenal masses (harmless fatty adrenal adenomas or more suspicious lesions). Diffusion-weighted imaging is included for detection of suspicious lesions like pheochromocytomas and NET. Dynamic series with intravenous contrast including the arterial phase T1 with fat suppression, allow detection of pheochromocytoma, hypervascular NET of the pancreas, and RCC.

**Fig. 3:**
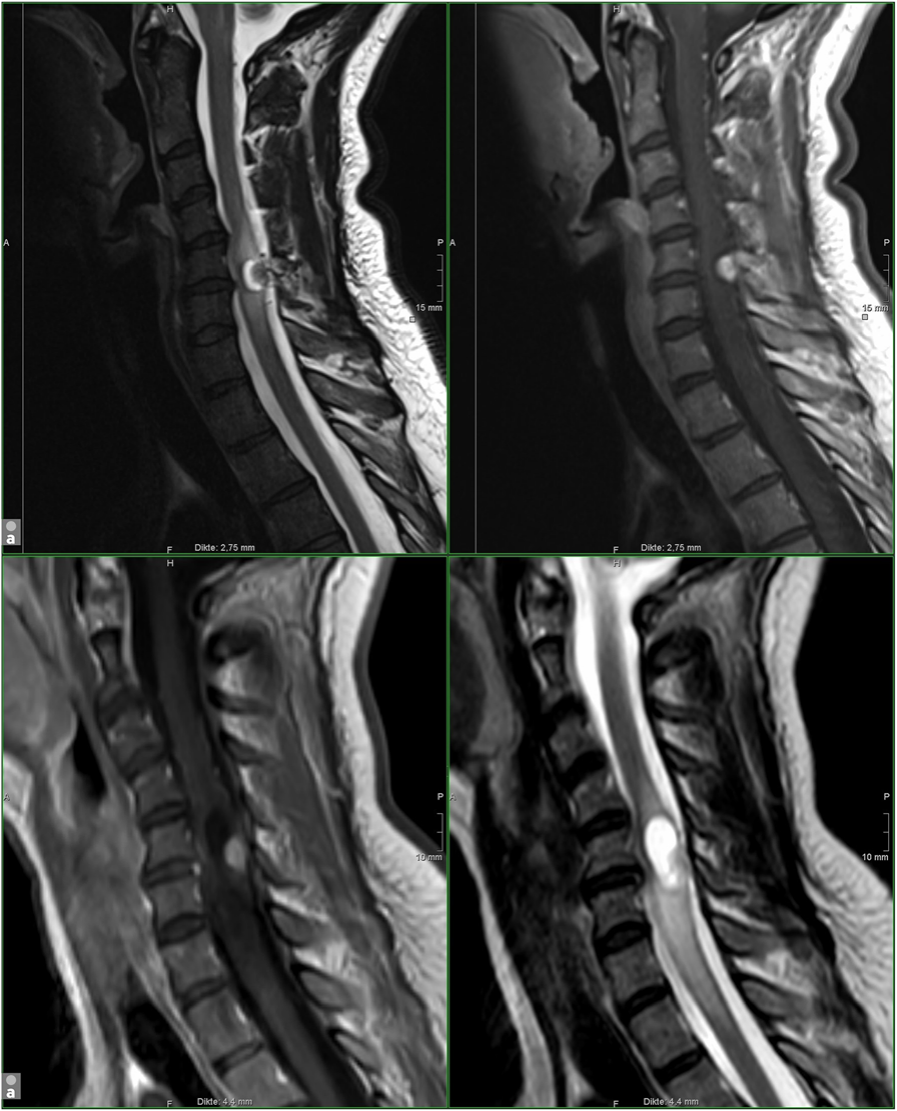
Spinal stage. Hemangioblastoma of the cervical spine in a 47-year-old woman with VHL disease. Upper-row (a): T1 - and T2 sagittal images of the cervical spine in our institution; routine spine protocol in July 2015. Lower-row (**b**): T1 - and T2 sagittal images of the cervical spine in our screening protocol in May 2017. HB consisting of a contrast enhancing nodule and a cyst with surrounding edema, C5-level; growing lesion. Uncomplicated surgery was performed

**Fig. 4:**
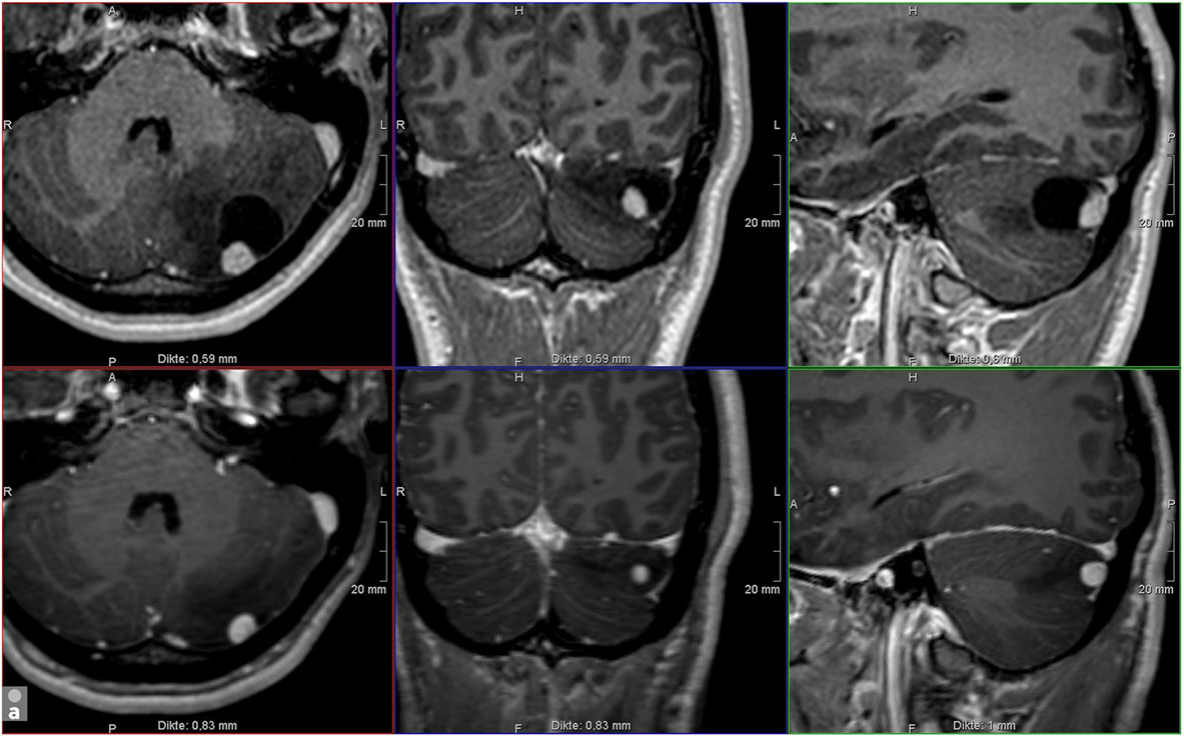
Cerebral stage. Hemangioblastoma in the left cerebellum in a 20-year-old male. Upper-row (**a**): 3D-T1WI with Gadolinium of our screening protocol in April 2018; Lower-row (**b**): 3D-T1WI with Gadolinium of an MRI brain in May 2017 in another institution. HB consisting of a contrast enhancing nodule and a cyst with surrounding edema. All three components clearly increase in dimension and are characteristic for a rapidly growing lesion. Although growth in HB is unpredictable and the patient had no complaints, given the localization, surgery was advised

After the series of the abdomen, series of the brain and spine are realized with the same Gd contrast, which was previously used for the abdomen. The cerebral and spinal sequences have a high sensitivity for detection of HB. We included sagittal T2 and T1 contrast-enhanced images for spinal cord lesions (Fig. 3), allowing to detect enhancing nodules, cysts, syrinx, aberrant vessels and edema in the spinal cord. We added axial 3D-FLAIR and sagittal 3D- FFE (fast field echo) -T1 post-contrast sequences for the brain lesions (Fig. 4), essentially depicting cystic/solid, enhancing or not enhancing posterior fossa lesions with or without surrounding edema, but also useful for ELST in the temporal bone. Occasionally, a retinal HB can be seen.

## Results

### Patients

From January 2016 to November 2018, we performed 38 scans in 18 VHL patients. In 13 patients, at least one follow-up scan after one year was available, as provided in the screening program. In the other 5 patients, a previous MRI of the brain, spine and abdomen was at our disposal from outside the institution. Because our combined protocol was tested in a tertiary center, most patients suffered from numerous lesions. In these 5 patients all lesions which were visible on the previous more extensive imaging, were also visible with the abbreviated protocol.

### Time

The calculated time needed to execute all the sequences of our MRI protocol is 27 min and 16 s (Fig. 1). The real MR examination time for our screening protocol is 35 min, including the necessary image reconstruction intervals.

### Detected lesions

14/18 patients are in follow-up for one or more hemangioblastomas in the posterior fossa and 2/18 for localization in the supratentorial region. One patient is followed for an HB of the optical nerve. 1/18 has an ELST and 1/18 patients is in follow-up for a possible ELST. Occasional retinal hemangioblastomas were visible (2/16).

11/18 patients had active lesions in the cervical spine and 11/18 in the thoracolumbar spine. 4/18 patients are in follow-up with a syrinx.

9/18 patients suffered from cystadenomas in the pancreas. 1/18 had a RCC in the left kidney (15 mm). 1/18 had a mass of uncertain behavior in the kidney, which is in follow-up. The RCC was treated with radiofrequency ablation (biopsy-proven diagnosis).

Numerous cysts in the kidneys and some in the liver were present. 1/18 had a harmless nodule in the adrenal gland and 3/18 had an adrenalectomy before the start of their screening in our institution. No one had suspicious lesions at the adrenals.

The one-step approach MRI examinations lead to 6 surgical interventions for radiologically progressive or symptomatic HB of the CNS. In that case, an MRI examination before the intervention and dedicated to the medical problem was planned. No unnecessary procedures, supplementary MRI nor other procedures were foreseen in the other patients. No one has contacted the hospital for clinically suspicious lesions, arisen in the time interval between two screening examinations. No lesions became symptomatic in the interval period between two screening sessions.

## Discussion

We report the first whole body MRI screening in patients with VHL disease.

Patients and families affected by hereditary tumor syndromes undergo periodic screening to enable timely preventive treatment of associated tumors. Almost annually, patients are subjected to multiple MRIs of different body regions, often scheduled on separate days. This approach results in screening fatigue and therapeutic non-compliance. Furthermore, the multitude of scans harbors financial consequences for both patient and national healthcare budget.

VHL patients typically develop various tumors in the brain, spine, and abdomen. We developed a one-step MRI-screening protocol for VHL patients, enabling clinicians to search for all relevant lesions on the common anatomical locations, in a shortened time frame of 35 min. A maximum of 15 mL of Gd was injected. During a study period of 3 years, we included 38 MRIs performed in 18 patients in our analysis. No lesions became symptomatic in the interval period of the screening.

Limited data exist on the use of whole-body MRI screening in oncology. Up to date, its primary application is monitoring disease status in cancers with high risk of widespread metastasis, such as multiple myeloma and melanoma [15]. To our great interest, whole-body MRI screening has recently been implemented in screening guidelines for two hereditary tumor syndromes: Li-Fraumeni and hereditary paraganglioma–pheochromocytoma syndrome [16–18]. No data exist on the use of whole-body MRI screening in VHL patients.

VHL patients appreciate the approach followed in our institution very well. The MRI protocol fits well in a one-day VHL clinical screening program, together with other standard recommended examinations (such as fundoscopy, audiogram, and urine catecholamine excretion measuring).

An advantage of our technique is the lower price due to the reduced number of MRI examinations and the use of a single dose of Gd contrast. Because most patients start screening early in life, and additional perioperative imaging is sometimes needed, limiting contrast exposure is of great importance. Renal tumor involvement and thus the risk for renal failure is not uncommon in VHL, and increases the risk for nephrogenic systemic sclerosis if using Gd contrast [19].

Since 2015, several authors have described CNS accumulation of Gd-based contrast agents [10, 11]. According to our previous research, some patient groups are even more prone to accumulation. We have proven a higher prevalence and rate of accumulation in VHL patients, than in patients affected by another hereditary tumor syndrome: tuberous sclerosis [12]. The difference in contrast deposition between various tumor syndromes may be explained by several factors, such as vascular leakage accompanying central nervous system hemangioblastomas.

Although long term clinical side-effects of accumulation are currently unknown, effective reduction of the number of contrast administrations seems understandable; especially if this can be accomplished by simple measures without sacrificing quality, such as our new focused MRI protocol.

## Conclusions

The developed one-step approach for imaging of the brain, spine, and abdomen in one setting (35 min) using a single dose of contrast, is - after evaluation in the last three years - experienced as a safe procedure for VHL disease screening.

## Data Availability

The datasets used and/or analyzed during the current study are not publicly available due to privacy matters but are available from the corresponding author on reasonable request.

## Abbreviations

CNS: Central nervous system
ELST: Endolymphatic sac tumor
FFE: Fast field echo
FLAIR: Fluid attenuated inversion recovery
Gd: Gadolinium
HB: Hemangioblastoma
MRI: Magnetic resonance imaging
NET: Neuroendocrine tumor
RCC: Renal cell carcinoma
T: Tesla
VHL: Von Hippel-Lindau
